# Economic and Clinical Impact of Stroke and Warfarin Use for Patients with Non-valvular Atrial Fibrillation

**DOI:** 10.36469/9852

**Published:** 2013-11-26

**Authors:** Li Wang, Elyse Fritschel, Onur Baser

**Affiliations:** 1 STATinMED Research, Dallas, TX, USA; 2 The University of Michigan and STATinMED Research, Ann Arbor, MI, USA

**Keywords:** clinical, economic, propensity score matching, cardiovascular, stroke, nvaf, retrospective

## Abstract

**Background:** Atrial fibrillation (AF) is a common clinical problem and potent risk factor for stroke. However, real-world effectiveness and outcomes for AF patients are not well described.

**Objective:** To compare the economic and clinical impact of stroke and warfarin use on patients with nonvalvular atrial fibrillation (NVAF).

**Methods:** This was a retrospective analysis of medical and pharmacy claims of NVAF patients from a large commercial health insurance database (01/01/2005-12/31/2007). Patients were grouped according to stroke or warfarin prescription status. For all groups, demographic, clinical, and pharmaceutical characteristics were analyzed descriptively. Risk-adjusted overall and cardiovascular-related hospital readmission rates in 30 days, length of stay (LOS), clinical outcomes, and health care costs were assessed using propensity score matching. Costs were adjusted to 2007 U.S. dollars using the medical component of the U.S. Consumer Price Index.

**Results:** Out of 18,575 NVAF patients, 3.1% (n=575) experienced a stroke event. Stroke patients were older on average (78.94 vs. 77.28 years, p-value<0.0001) with significantly higher risk-adjusted inpatient mortality (7.14% vs. 2.09%, p-value<0.0001), emergency room visits (79.97% vs. 46.34%, p-value<0.0001), and average LOS measures (overall: 10.20 vs. 6.83 days, p<0.0001; cardiovascular-related: 8.35 vs. 5.90 days, p-value<0.0001). Despite the similarity in Charlson Comorbidity Index scores compared to non-stroke controls, stroke patients significantly higher clinical outcome rates during follow-up for acute coronary syndrome (ACS), ischemic attack, major and minor bleeding patients (p-values<0.0100), and the total cost incurred was nearly three times greater ($33,506 vs. $13,921, p-values<0.0001). NVAF patients were commonly prescribed warfarin (65.60%) and appeared to have a lower prevalence of clinical outcomes, while not incurring significantly higher follow-up costs compared to those not prescribed warfarin ($12,739, standard deviation [SD]=$19,842 vs. $15,358; SD=$45,446; p-value>0.0500). However, a significantly greater proportion of patients with major and minor bleeding were prescribed a combination of warfarin and antiplatelets than those without these events.

**Conclusions:** A stroke after an NVAF diagnosis has a major clinical impact, which translates into a significant economic burden for patients. Warfarin prescriptions did not significantly impact total health care costs, though caution is advised to minimize hemorrhagic events.

## INTRODUCTION

Atrial fibrillation (AF) is the most common significant cardiac arrhythmia, affecting an estimated 2.3 million adults in the United States. Its prevalence increases with age, approximately doubling each decade in individuals over age 50 years. In those over age 65 years, AF prevalence is estimated to be 6%[1] and roughly 10% in persons age 80 years or older.[2,3]

AF patients have six times the risk of ischemic stroke, due to cardiogenic embolism, than age-matched controls, resulting in approximately 80,000 strokes per year in North America.[4] The risk of stroke in AF patients increases with advancing age and the presence of concomitant risk factors including left ventricular dysfunction, history of hypertension, stroke or transient ischemic attack (TIA), and diabetes mellitus (DM).[5] Medical therapy has been shown to ameliorate several complications associated with AF, particularly with regard to reducing stroke risk.[6] Among appropriate diagnosed AF patients, anticoagulation can reduce the risk of a first stroke by approximately 68%,[7] and it is the recommended therapy in published guidelines.[8] Therefore, an expert panel has recommended that all AF patients over age 75 should be considered for chronic anticoagulation therapy, unless a contraindication exists.

Elderly patients with an ischemic stroke associated with AF are at especially high risk for a recurrent stroke with an annual rate of more than 10% per year.[9] Secondary preventative therapy with warfarin is highly effective in reducing this risk. The recurrence rate can be reduced by two thirds with warfarin therapy. Warfarin use was also confirmed to decrease mortality.[10]

AF is a common clinical problem and potent risk factor for stroke, yet there is a lack of studies focusing on the real-world effectiveness and safety of outcomes for AF patients.

## METHODS

### Study Sample

This study used data from a large U.S. commercial database from 2003 to 2008. Table 1 represents patient attrition. The prevalence of stroke was analyzed in selected patients aged 65 years or older with claims for at least two primary diagnoses for AF (determined using the International Classification of Diseases 9$^{th}$ Revision Clinical Modification [ICD-9-CM] codes in Appendix Table A1) occurring within 30 days of one another. Additional criteria for study selection included continuous health plan enrollment for at least 180 days prior to the initial AF diagnosis date (designated as the index date), and the earlier date of at least 180 days following the first AF diagnosis date or until death.

Patients were excluded if they had at least one medical claim for any the following: transient AF caused by a reversible disorder, known presence of atrial myxoma, left ventricular thrombus, active endocarditis, any diagnosis of arterial or venous thromboembolism (VTE), use of selected antiplatelet or anticoagulant agents (low-molecular-weight [LMWH] and unfractionated heparin [UFH], fondaparinux, anisindione, abciximab, cilostazol, anagrelide, pentoxifylline, enoxaparin, dalteparin, or clopidogrel), before the first AF diagnosis. Patients must have had continuous enrollment for at least 180 days before and after the first prescription date. From this final sample (n=19,268), cohorts were assigned; patients were assigned to appropriate cohorts.

**Table 1.** Attrition Table

| Condition | Sample Size |
|---|---|
| Initial Sample Size | 269,339 |
| 2 or more AF diagnoses occurring within 30 days of one another | 145,938 |
| Age 65 years and older (according to first AF diagnosis date) | 140,913 |
| Continuous enrollment for at least 180 days pre-first AF diagnosis date and at least 180 days following first AF diagnosis date or until death, whichever comes first | 90,319 |
| AF, atrial myxoma, left ventricular thrombus, active endocarditis, any diagnosis of arterial or VTE, planned cardioversion or trauma diagnose before first AF diagnosis (index) date were excluded. | 23,373 |
| Patients prescribed selected antiplatelet or anticoagulant agents (dalteparin, enoxaparin, tinzaparin, heparin sodium in dextrose, heparin sodium, heparin sodium injection, heparin sodium/sodium chloride, fondaparinux, anisindione, abciximab, cilostazol, anagrelide, pentoxifylline) before first diagnosis date were excluded. | 19,702 |
| Continuous enrollment for at least 180 days prior to first prescription date and at least 180 days following first prescription date. | 19,268 |
| Diagnosed with NVAF | 18,575 |

AF=atrial fibrillation; VTE=venous thromboembolism; NVAF=non-valvular atrial fibrillation

Non-valvular AF (NVAF) patients did not have pre-index claims indicating the presence of transient, perioperative AF, valvular heart disease or concomitant hyperthyroidism or prescriptions for methimazole or propylthiouracil.

### Cohort Assignment

NVAF patients were stratified into the following cohorts based on follow-up criteria:

*Warfarin or Non-warfarin Cohorts:* Patients included in the Warfarin Cohort had at least one prescription for warfarin or dicumarol after the index date, more than one outpatient international normalized ratio (INR) (current procedural terminology [CPT] code: 99211), or a diagnosis or warfarin therapy in the 3 months after initial identified AF diagnosis during the study period, or any combination of these factors. The date of whichever came first was designated as the drug index date; and

*Stroke or Non-stroke Cohorts:* Stroke Cohort patients had at least one claim for an ischemic or hemorrhagic stroke in the follow-up period (ICD-9-CM codes: 430.xx, 431.xx, 432.xx, 433.xx, 434.xx, 436.0).

## COVARIATES AND OUTCOME VARIABLES

### Patient Characteristics

Many aspects of health care utilization and cost, including treatment selection, therapy patterns, and health outcomes, may be associated with factors not directly measured in administrative claims data. Vast literature exist demonstrating differences in a variety of health-related outcomes for patients of differing educational attainment, income, net worth, race and ethnicity and family structure. Therefore, various patient and clinical factors to estimate risk-adjusted comparisons were captured.

Age was defined as of the index date. In addition, patient age was used to assign them to the following age groups: 65-74, 75-84, 85-94, 95+. Median and maximum values were also provided. Gender and U.S. region variables (Northeast, North Central, South, and West) were included as well. The most commonly used comorbidity index in health outcomes studies is the Charlson Comorbidity Index (CCI), which assigns a weight ranging from 1 to 6 according to disease severity for 19 conditions.[11] The index has since adopted several weights, some of which allow outpatient diagnoses to contribute to the score.[12,13]

### Comorbid Conditions

The Appendix Table lists disease codes for comorbid conditions and clinical outcomes measured for the baseline period (≥180 days pre-index date). Patients were flagged for the following conditions: end stage renal disease (ESRD), congestive heart failure (CHF), peripheral arterial disease (PAD), acute coronary syndrome (ACS), hyperthyroidism, obesity, DM, hypertension, ischemic stroke, hemorrhagic stroke, non-central nervous system (CNS) systemic embolism, TIA, catheter ablation, and dyspepsia. Studied medications included warfarin, injectable anticoagulants (enoxaparin, dalteparin, and fondaparinux), antiplatelets (plavix), and combinations thereof.

### Outcome Variables

A flag was created for each of the following variables occurring after the index date (Table 2): in-hospital mortality, average number of health care visits per patient per year (pppy), readmission rates 30 days after discharge, length of stay (LOS) (all-cause and cardiovascular-related), ACS, ischemic stroke, hemorrhagic stroke, TIA, major bleeding, non-major clinically relevant bleeding, non-CNS systemic embolism, surgical ablation, cardioversion, intra-cardiac device, myocardial infarction (MI), osteopathic fracture, intracranial hemorrhage (ICH), and gastrointestinal (GI) bleeding.

Average total annual health care costs per patient were captured as well. Since the analysis is from the payer's perspective, reimbursement amounts rather than charges were applied. Expenditures were expressed in 2007 U.S. dollars and were adjusted using the medical component of the U.S. Consumer Price Index.

## STATISTICAL ANALYSIS

Baseline and outcome measures were descriptively analyzed using numbers/percentages for dichotomous and means/standard deviation (SD) for continuous variables. Bivariate comparisons were implemented using t-tests or chi-square tests when appropriate with standardized differences included. Non-parametric tests (e.g. the Mann Whitney U-test) were applied if there was a deviation from asymptotical assumptions.

Multivariate analysis was performed using 1:1 propensity score matching (PSM). Propensity scores were estimated via unconditional logistic regression analysis to yield risk-adjusted estimates (or remove overt bias). Patients were matched if their propensity scores were within ±0.01 units of one another. Covariates in the logistic regression model included age, gender, baseline CCI score, baseline comorbid conditions, baseline health care utilization, and baseline health care costs. To estimate total cost, a generalized linear model (GLM) with gamma distribution and log link function with cost as the dependent variable and demographic/clinical factors used in PSM as independent variables.

**Table 2.** Descriptive Statistics for NVAF Patients who did or did not Experience a Stroke

| | NVAF Patients (N=18,575) | | | | | |
|---|---|---|---|---|---|---|
| | No Stroke (N=18,000) | | Stroke (N=575) | | | |
| | Mean/N | SD/% | Mean/N | SD/% | P-value | Std Diff |
| **Pre-period (180 days before AF diagnosis date)** | | | | | | |
| **Age (mean)** | 77.28 | 7.12 | 78.94 | 6.77 | 0 | 23.99 |
| **Median** | 77 | | 79 | | | |
| **Maximum** | 103 | | 96 | | | |
| **65-74** | 6788 | 37.71% | 152 | 26.43% | 0 | 24.33 |
| **75-84** | 8161 | 45.34% | 295 | 51.30% | 0.0047 | 11.95 |
| **85-94** | 2911 | 16.17% | 124 | 21.57% | 0.0006 | 13.81 |
| **95+** | 140 | 0.78% | 4 | 0.70% | 0.8251 | 0.96 |
| **Gender (Male)** | 8852 | 49.18% | 275 | 47.83% | 0.5233 | 2.7 |
| **Region** | | | | | | |
| **Northeast** | 1892 | 10.51% | 50 | 8.70% | 0.1613 | 6.16 |
| **North Central** | 6372 | 35.40% | 228 | 39.65% | 0.036 | 8.79 |
| **South** | 4999 | 27.77% | 165 | 28.70% | 0.6266 | 2.05 |
| **West** | 4720 | 26.22% | 131 | 22.78% | 0.0645 | 8 |
| **Comorbid Conditions** | | | | | | |
| **Elixhauser Index Score (≥2%)** | 0.06 | 0.24 | 0.05 | 0.21 | 0.1697 | 5.52 |
| **CCI Score** | 0.19 | 0.7 | 0.22 | 0.81 | 0.3459 | 4.25 |
| **ESRD** | 45 | 0.25% | 4 | 0.70% | 0.0403 | 6.5 |
| **CHF** | 697 | 3.87% | 13 | 2.26% | 0.0473 | 9.35 |
| **PAD** | 27 | 0.15% | 3 | 0.52% | 0.0289 | 6.42 |
| **ACS** | 442 | 2.46% | 11 | 1.91% | 0.4064 | 3.71 |
| **Obesity** | 10 | 0.06% | 1 | 0.17% | 0.2508 | 3.49 |
| **Diabetes** | 299 | 1.66% | 9 | 1.57% | 0.8593 | 0.76 |
| **Hypertension** | 636 | 3.53% | 17 | 2.96% | 0.4597 | 3.25 |
| **Ischemic Stroke** | 335 | 1.86% | 30 | 5.22% | 0 | 18.23 |
| **Hemorrhagic Stroke** | 19 | 0.11% | 6 | 1.04% | 0 | 12.42 |
| **TIA** | 120 | 0.67% | 4 | 0.70% | 0.933 | 0.35 |
| **Catheter Ablation** | 6 | 0.03% | 0 | 0.00% | 0.6615 | 2.58 |
| **Dyspepsia** | 6 | 0.03% | 0 | 0.00% | 0.6615 | 2.58 |
| **Pharmacotherapy Pattern (AF diagnosis date – June 2007)** | | | | | | |
| **Warfarin** | 11818 | 65.66% | 368 | 64.00% | 0.4107 | 3.47 |
| **Injectable Anticoagulant** | 144 | 0.80% | 2 | 0.35% | 0.2268 | 5.99 |
| **Antiplatelet** | 545 | 3.03% | 44 | 7.65% | 0 | 20.67 |
| **Other Drug** | 4764 | 26.47% | 134 | 23.30% | 0.0902 | 7.32 |
| **Other Drug (Antiarrhythmic)** | 560 | 3.11% | 19 | 3.30% | 0.793 | 1.1 |
| **Other Drug(Rate Control Drugs)** | 2008 | 11.16% | 61 | 10.61% | 0.6816 | 1.76 |
| **No Medication** | 729 | 4.05% | 27 | 4.70% | 0.4405 | 3.16 |
| **Warfarin and Anticoagulant** | 793 | 4.41% | 37 | 6.43% | 0.0204 | 8.97 |
| **Warfarin and Antiarrhythmic** | 3358 | 18.66% | 117 | 20.35% | 0.3057 | 4.27 |
| **Warfarin and Antiplatelets** | 516 | 2.87% | 29 | 5.04% | 0.0023 | 11.18 |
| **Warfarin and Rate Control** | 9691 | 53.84% | 300 | 52.17% | 0.4305 | 3.33 |
| **Warfarin and Antidepressants** | 399 | 2.22% | 20 | 3.48% | 0.0449 | 7.59 |

NVAF=non-valvular atrial fibrillation; SD=standard deviation; AF=atrial fibrillation; CCI=Charlson comorbidity index ESRD=end-stage renal disease; CHF=congestive heart failure; PAD=peripheral arterial disease; ACS=acute coronary syndrome; TIA=transient ischemic attack

Multivariate analysis was performed using 1:1 propensity score matching (PSM). Propensity scores were estimated via unconditional logistic regression analysis to yield risk-adjusted estimates (or remove overt bias). Patients were matched if their propensity scores were within ±0.01 units of one another. Covariates in the logistic regression model included age, gender, baseline CCI score, baseline comorbid conditions, baseline health care utilization, and baseline health care costs. To estimate total cost, a generalized linear model (GLM) with gamma distribution and log link function with cost as the dependent variable and demographic/clinical factors used in PSM as independent variables.

## RESULTS

### Stroke vs. Non-stroke Cohorts

Of all NVAF patients, 3.10% experienced a stroke during the study period (n=575, Table 2). These stroke-experienced patients were older on average (78.94 vs. 77.28 years, p-value<0.0001) and had higher frequencies of ESRD (0.70% vs. 0.25%, p-value=0.040), PAD (0.52% vs. 0.15%, p-value=0.029), but lower frequency of CHF (2.26% vs. 3.87%, p-value=0.047) relative to those who did not suffer a stroke prior to NVAF diagnosis. Stroke-experienced patients were more frequently prescribed antiplatelets (7.65% vs. 3.03%, p-value<0.0001) or warfarin combinations with either anticoagulants (6.43% vs. 4.41%, p-value=0.020) or antiplatelets (5.04% vs. 2.87%, p-value=0.002) compared to non-stroke patients.

After PSM (Table 3), stroke patients had significantly higher risk-adjusted rates of inpatient mortality (7.14% vs. 2.09%, p-value<0.0001), emergency room (ER) visits (79.97% vs. 46.34%, p-value<0.0001), and average LOS measures (overall: 10.20 vs. 6.83 days, p-value<0.0001; cardiovascular-related: 8.35 vs. 5.90 days, p-value<0.0001). Significantly higher average clinical outcome rates per 100 pppy also occurred for the following conditions: ACS (14.98 vs. 5.57, p-value=0.0003), TIA (25.44 vs. 1.39, p-value<0.0001), major bleeding (29.62 vs. 1.39, p-value<0.0001) and minor bleeding (18.82 vs. 4.18, p-value<0.0001). Many post-index period comorbid conditions (ESRD, CHF, PAD, DM, hypertension) were more common in stroke patients as well. Consistent with the high adverse event rates and complex comorbidity profile, patients who experienced a stroke had health care costs nearly three times greater than patients who did not experience a stroke ($33,506 vs. $13,921, p-value<0.0001).

**Table 3.** Risk-adjusted Outcomes for NVAF Patients who did or did not Experience Stroke

| | After Match N=574 for Each Group | | | | | |
|---|---|---|---|---|---|---|
| | No Stroke | | Stroke | | | |
| | Mean/N | SD/% | Mean/N | SD/% | P-value | Std Diff |
| **Mortality-Inpatient** | 12 | 2.09% | 41 | 7.14% | <0.0001 | 24.23 |
| **# Patients with Inpatient Visit** | 255 | 44.43% | 574 | 100.00% | <0.0001 | 158.04 |
| **# Patients with ER Visit** | 266 | 46.34% | 459 | 79.97% | <0.0001 | 74.30 |
| **# Patients with Outpatient Visit** | 541 | 94.25% | 532 | 92.68% | 0.2786 | 6.34 |
| **# Patients with INR Visit** | 143 | 24.91% | 111 | 19.34% | 0.0223 | 13.45 |
| **Readmission Rate (30 days)** | 0 | 0.00% | 10 | 1.74% | <0.0001 | 18.81 |
| **LOS** | 7.11 | 10.7 | 10.05 | 12.25 | <0.0001 | 25.60 |
| **Cardiovascular-related** | | | | | | |
| **Readmission Rate (30 days)** | 0 | 0.00% | 10 | 1.74% | <0.0001 | 18.81 |
| **LOS** | 5.9 | 6.98 | 8.35 | 9.53 | <0.0001 | 29.34 |
| **Clinical Outcomes (100 person-years)** | | | | | | |
| **ACS** | 16 | 5.57 | 43 | 14.98 | 0.0003 | 21.41 |
| **TIA** | 4 | 1.39 | 73 | 25.44 | <0.0001 | 49.46 |
| **Major Bleeding** | 4 | 1.39 | 85 | 29.62 | <0.0001 | 54.66 |
| **Non-major Clinical Relevant Bleeding** | 12 | 4.18 | 54 | 18.82 | <0.0001 | 31.80 |
| **Non-CNS Systemic Embolism** | 4 | 1.39 | 8 | 2.79 | 0.2482 | 6.85 |
| **Stroke or Non-CNS Systemic Embolism** | 4 | 1.39 | 574 | 200.00 | <0.0001 | 1686.72 |
| **Catheter Ablation** | 2 | 0.70 | 4 | 1.39 | 0.4142 | 4.83 |
| **PCI** | 2 | 0.70 | 2 | 0.70 | 1.0000 | 0.00 |
| **CABG Surgery** | 1 | 0.35 | 13 | 4.53 | 0.0013 | 19.12 |
| **MI** | 9 | 3.14 | 32 | 11.15 | 0.0002 | 21.70 |
| **Osteoporotic Fracture** | 4 | 1.39 | 16 | 5.57 | 0.0073 | 16.02 |
| **Dyspepsia** | 0 | 0.00 | 3 | 1.05 | <0.0001 | 10.24 |
| **ICH** | 0 | 0.00 | 77 | 26.83 | <0.0001 | 55.62 |
| **GI Bleeding** | 3 | 1.05 | 7 | 2.44 | 0.1573 | 7.50 |
| **Health Care Cost** | | | | | | |
| **Total Health Care Costs** | $16,375 | $65,996 | $33,430 | $55,050 | <0.0001 | 28.06 |

SD=standard deviation; INR=international normalized ratio; LOS=length of stay; ACS=acute coronary syndrome; TIA=transient ischemic attack; CNS=central nervous system; PCI=percutaneous coronary intervention; CABG=coronary artery bypass graft; MI=myocardial infarction; ICH=intracranial hemorrhage; GI=gastrointestinal

## NVAF Patients with/without Warfarin Prescription

Among 18,575 patients with NVAF, 12,816 were included in the Warfarin Cohort and 6,389 were in the Non-warfarin Cohort (Table 4). The Warfarin Cohort was relatively young (76.64 vs. 78.65 years, p-value<0.0001) and healthy, with a lower CCI index score (0.15 vs. 0.27, p-value<0.0001) and lower proportion of patients with nearly all comorbid conditions except hyperthyroidism, obesity, non-CNS systemic embolism, catheter ablation, or dyspepsia (p-values>0.0500). The resulting unadjusted total health care costs were lower for patients prescribed warfarin ($13,906, SD=$27,285 vs. $15,712, SD=$44,832; p-value=0.0033). Since these measures are confounded with baseline characteristics, PSM was applied.

**Table 4.** Descriptive Statistics for NVAF Patients Prescribed Warfarin or Not

| | NVAF Patients (N=18,575) | | | | | |
|---|---|---|---|---|---|---|
| | Non-warfarin Cohort (N=6,389) | | Warfarin Cohort (N=12,186) | | | |
| | Mean/N | SD/% | Mean/N | SD/% | P-value | Std Diff |
| **Pre-period (180 days pre-AF diagnosis date)** | | | | | | |
| **Age (mean)** | 78.65 | 7.77 | 76.64 | 6.64 | 0 | 27.8 |
| **Median** | 79 | | 76 | | | |
| **Maximum** | 102 | | 103 | | | |
| **65-74** | 2,103 | 32.92% | 4,837 | 39.69% | 0 | 14.13 |
| **75-84** | 2,699 | 42.24% | 5,757 | 47.24% | 0 | 10.06 |
| **85-94** | 1,480 | 23.16% | 1,555 | 12.76% | 0 | 27.35 |
| **95+** | 107 | 1.67% | 37 | 0.30% | 0 | 13.89 |
| **Gender (Male)** | 2,864 | 44.83% | 6,263 | 51.40% | 0 | 13.17 |
| **Region** | | | | | | |
| **Northeast** | 654 | 10.24% | 1,288 | 10.57% | 0.4808 | 1.09 |
| **North Central** | 2,217 | 34.70% | 4,383 | 35.97% | 0.0865 | 2.65 |
| **South** | 1,808 | 28.30% | 3,356 | 27.54% | 0.2728 | 1.69 |
| **West** | 1,701 | 26.62% | 3,150 | 25.85% | 0.2536 | 1.76 |
| **Comorbid Conditions** | | | | | | |
| **CCI Score** | 0.27 | 0.86 | 0.15 | 0.61 | 0 | 16.66 |
| **ESRD** | 27 | 0.42% | 22 | 0.18% | 0.0022 | 4.42 |
| **CHF** | 300 | 4.70% | 410 | 3.36% | 0 | 6.77 |
| **PAD** | 18 | 0.28% | 12 | 0.10% | 0.0031 | 4.21 |
| **ACS** | 179 | 2.80% | 274 | 2.25% | 0.0202 | 3.53 |
| **Obesity** | 3 | 0.05% | 8 | 0.07% | 0.6189 | 0.79 |
| **Diabetes** | 131 | 2.05% | 177 | 1.45% | 0.0024 | 4.56 |
| **MI** | 93 | 1.46% | 120 | 0.98% | 0.0042 | 4.29 |
| **Hypertension** | 296 | 4.63% | 357 | 2.93% | 0 | 8.94 |
| **Ischemic Stroke** | 159 | 2.49% | 206 | 1.69% | 0.0002 | 5.58 |
| **Hemorrhagic Stroke** | 20 | 0.31% | 5 | 0.04% | 0 | 6.47 |
| **TIA** | 56 | 0.88% | 68 | 0.56% | 0.0113 | 3.77 |
| **Catheter Ablation** | 0 | 0.00% | 6 | 0.05% | 0.0761 | 3.14 |
| **Dyspepsia** | 1 | 0.02% | 5 | 0.04% | 0.3605 | 1.51 |
| **Pharmacotherapy Pattern (AF diagnosis date - June 2007)** | | | | | | |
| **Warfarin** | 0 | 0.00% | 12,186 | 100.00% | 0 | 0 |
| **Injectable Anticoagulant** | 146 | 2.29% | 0 | 0.00% | 0 | 21.63 |
| **Antiplatelet** | 589 | 9.22% | 0 | 0.00% | 0 | 45.06 |
| **Other Drug** | 4,898 | 76.66% | 0 | 0.00% | 0 | 256.3 |
| **Other Drug (Rate Control Drugs)** | 2,069 | 32.38% | 0 | 0.00% | 0 | 97.86 |
| **No Medication** | 756 | 11.83% | 0 | 0.00% | 0 | 51.81 |
| **Warfarin and Anticoagulant** | 0 | 0.00% | 830 | 6.81% | 0 | 38.23 |
| **Warfarin and Antiarrhythmic** | 0 | 0.00% | 3,475 | 28.52% | 0 | 89.32 |
| **Warfarin and Antiplatelets** | 0 | 0.00% | 545 | 4.47% | 0 | 30.6 |
| **Warfarin and Rate Control** | 0 | 0.00% | 9,991 | 81.99% | 0 | 301.71 |

NVAF=non-valvular atrial fibrillation; SD=standard deviation; AF=atrial fibrillation; CCI=Charlson comorbidity index; ERD=end-stage renal disease; CHF=congestive heart failure; PAD=peripheral arterial disease; ACS=acute coronary syndrome; MI=myocardial infarction; TIA=transient ischemic attack

After matching, the adjusted outcomes of 5,966 warfarin and non-warfarin control patients were compared. Warfarin patient in-hospital mortality (0.75% vs. 2.26%, p-value<0.0001) and ER visit rates (48.79% vs. 53.30%, p-value<0.0001) and average cardiovascular-related hospital LOS (5.81 vs. 5.84 days, p-value<0.0001) were lower than those of the matched controls (Table 5). While the rates of clinical outcomes per 100 pppy were generally similar, warfarin patients had lower rates of ACS (3.52 vs. 5.16, p-value=0.0022) and MI (2.04 vs. 3.75, p-value<0.0001) and a higher rate of TIA (2.15 vs. 1.41, p-value=0.033) compared to non-warfarin patients. The resulting health care costs for warfarin and non-warfarin patients was similar after risk adjustment ($12,739, SD=$19,842 vs. $15,358, SD=$45,446; p-value>0.0500). In the post-index period, warfarin patients had higher follow-up rates of CHF (21.25 vs. 17.40, p-value=0.0003) but lower rates of ESRD (0.64 vs. 1.64, p-value=0.0003).

**Table 5.** Risk-adjusted Outcomes for NVAF Patients Prescribed Warfarin or Not

| | After Match N=5,966 for each Cohort | | | | | |
|---|---|---|---|---|---|---|
| | Non Warfarin Cohort | | Warfarin Cohort | | | |
| | Mean/N | SD/% | Mean/N | SD/% | P-value | SD |
| **Mortality-Inpatient** | 135 | 2.26% | 45 | 0.75% | <0.0001 | 12.4 |
| **# Patients with Inpatient Visit** | 2615 | 43.83% | 2706 | 45.36% | 0.0913 | 3.07 |
| **# Patients with ER Visit** | 3180 | 53.30% | 2911 | 48.79% | <0.0001 | 9.03 |
| **# Patients with Outpatient Visit** | 5428 | 90.98% | 5842 | 97.92% | <0.0001 | 30.67 |
| **# Patients with INR Visit** | 340 | 5.70% | 2197 | 36.83% | <0.0001 | 82.25 |
| **Readmission Rate (30 days)** | 22 | 0.37% | 18 | 0.30% | 0.5271 | 1.16 |
| **LOS** | 7.76 | 13.88 | 6.54 | 7.92 | 0.066 | 10.75 |
| **Cardiovascular-related** | | | | | | |
| **Readmission Rate (30 days)** | 12 | 0.20% | 16 | 0.27% | 0.4497 | 1.39 |
| **LOS** | 5.84 | 8.51 | 5.81 | 6.1 | <0.0001 | 0.47 |
| **Clinical Outcomes (100 person-years)** | | | | | | |
| **ACS** | 154 | 5.16 | 105 | 3.52 | 0.0022 | 5.64 |
| **Ischemic Stroke** | 167 | 5.6 | 178 | 5.97 | 0.5502 | 1.1 |
| **Hemorrhage Stroke** | 33 | 1.11 | 23 | 0.77 | 0.1814 | 2.45 |
| **TIA** | 42 | 1.41 | 64 | 2.15 | 0.0326 | 3.93 |
| **Major Bleeding** | 75 | 2.51 | 88 | 2.95 | 0.3026 | 1.88 |
| **Non-major Clinical Relevant Bleeding** | 148 | 4.96 | 137 | 4.59 | 0.5102 | 1.21 |
| **Non-CNS Systemic Embolism** | 37 | 1.24 | 48 | 1.61 | 0.2328 | 2.19 |
| **Stroke or Non-CNS Systemic Embolism** | 225 | 7.54 | 234 | 7.84 | 0.6703 | 0.78 |
| **Catheter Ablation** | 25 | 0.84 | 38 | 1.27 | 0.1015 | 3.01 |
| **PCI** | 45 | 1.51 | 29 | 0.97 | 0.0593 | 3.42 |
| **CABG Surgery** | 14 | 0.47 | 9 | 0.3 | 0.2971 | 1.91 |
| **MI** | 112 | 3.75 | 61 | 2.04 | <0.0001 | 7.16 |
| **Osteoporotic Fracture** | 65 | 2.18 | 45 | 1.51 | 0.0565 | 3.51 |
| **Dyspepsia** | 5 | 0.17 | 2 | 0.07 | 0.2568 | 2.08 |
| **ICH** | 37 | 1.24 | 26 | 0.87 | 0.1658 | 2.54 |
| **GI Bleeding** | 32 | 1.07 | 49 | 1.64 | 0.0589 | 3.47 |
| **Health Care Costs** | | | | | | |
| **Total Health Care Costs** | $15,359 | $45,446 | $12,739 | $19,842 | 0.3704 | 7.47 |

SD=standard deviation; INR=international normalized ratio; LOS=length of stay; ACS=acute coronary syndrome; TIA=transient ischemic attack; CNS=central nervous system; PCI=percutaneous coronary intervention; CABG=coronary artery bypass graft; MI=myocardial infarction; ICH=intracranial hemorrhage; GI=gastrointestinal

### NVAF Patient Pharmacotherapy Patterns with Adverse Outcomes

NVAF patients with major bleeding (n=266) were prescribed warfarin plus antiplatelet combinations (6.02% vs. 2.89%, p-value=0.0027) significantly more often than those without major bleeding (n=18,309) (Table 6). Similarly, when NVAF patients with non-major bleeding (n=454) were compared to controls lacking non-major bleeding (n=18,121), more non-major bleeding patients were prescribed warfarin+antiplatelets after an AF diagnosis (4.63% vs. 2.89%, p-value=0.0306).

NVAF patients diagnosed with GI bleeding (n=145) were prescribed warfarin significantly more often than the 18,430 patients without GI bleeding (74.48% vs. 65.53%, p-value=0.0239). However, no significant difference was found when comparing warfarin+antiplatelet prescriptions (5.52% vs. 2.91%, p-value=0.0643). NVAF patients who experienced ICH (n=84) were prescribed warfarin alone less often than the 18,491 patients without ICH (53.57% vs. 65.66%, p-value=0.0200), and despite a higher absolute frequency of ICH patients with warfarin+antiplatelet prescriptions (5.95% vs. 2.92%, p-value=0.1004), the difference was not statistically significant.

**Table 6.** Pharmacotherapy Patterns for Patients with Bleeding/Hemmorhagic Outcomes after AF Diagnosis

| Non-major Bleeding (%) | | | GI Bleeding (%) | | | ICH (%) | | |
|---|---|---|---|---|---|---|---|---|
| Yes (n=454) | No (n=18,121) | P-value | Yes (n=145) | No (n=18,430) | P-value | Yes (n=84) | No (n=18,491) | P-value |
| 63.66 | 65.65 | 0.3764 | 74.48 | 65.53 | 0.0239 | 53.57 | 65.66 | 0.02 |
| 4.63 | 2.89 | 0.0306 | 5.52 | 2.91 | 0.0643 | 5.95 | 2.92 | 0.1004 |

GI=gastrointestinal; ICH=intracranial hemmorhage

## DISCUSSION

Stroke in NVAF patients is a clinically relevant event with increased spending implications in Medicare Supplemental patients. Warfarin was commonly prescribed to NVAF patients without a significant increase in total health care expenses. Consideration of the dose intensity of warfarin may help avoid hemorrhagic complications.

An established adverse event associated with warfarin use is hemorrhage, particularly when in combination with antiplatelets.[14,15] Observational data from this study generally support this claim; a significantly greater proportion of patients with major and minor bleeding were prescribed a combination of warfarin and antiplatelets than those without these events. Absolute frequencies of warfarin and antiplatelet use were higher in GI bleeding and ICH patients, though their small sample sizes may have contributed to the lack of statistical significance, compared to controls without these conditions. These considerations should be kept in mind when creating an optimal treatment plan for NVAF patients.

A major strength of this study involves the use of a large database of nationwide, physician-designated hospital diagnoses with real-world spending figures from a recent time period. While claims data are extremely valuable for the efficient and effective examination of health care outcomes, treatment patterns, and costs, claims data are collected for the purpose of payment and not research. Therefore, there are certain limitations associated with the use of claims data. The presence of a claim for a filled prescription does not indicate that the medication was consumed or that it was taken as prescribed. Additionally, the presence of a diagnosis code on a medical claim is not positive presence of disease, as the diagnosis code may be incorrectly coded or included as rule-out criteria rather than actual disease. Finally, certain information is not readily available in claims data that could have an effect on study outcomes, such as clinical and disease-specific parameters.

It should be noted that neither regression adjustment addresses problems due to imbalances in unmeasured factors. It is quite possible that outcomes for patients with the same observable characteristics can vary because of unobservable factors such as physician or practice-prescribing patterns. There are several methods that exist to control for unmeasured factors such as the instrumental variable approach, bounding approach and a difference-in-difference estimator. However, these estimators are also confounded by their own limitations.

## CONCLUSIONS

A stroke after an NVAF diagnosis has a major clinical and economic impact on patients. Stroke-experienced patients had higher average clinical outcome rates than non-stroke controls as well as more frequent health care resource use (ER visits and longer average hospital LOS) and higher inpatient mortality. These factors contributed to annual total health care costs for stroke-experienced patients nearly three times greater than for those who did not experience a stroke.

Stroke incidence is nearly 5-fold higher in AF patients, so diagnosed patients must be monitored carefully and provided with appropriate pharmacotherapy to avert stroke events.[16,17] NVAF patients were commonly prescribed warfarin and appear to have a lower prevalence of clinical outcome conditions, while not incurring significantly higher follow-up costs compared to those not prescribed warfarin.

It should be noted that warfarin patients were also younger than controls on average and had a less complex comorbidity profile than patients without a warfarin prescription. Additional research would be necessary to confirm and explain the real-world outcomes seen in this population in a causative manner.

## CONFLICT OF INTEREST DECLARATION

The authors declare that they have no competing interests.

## Figures and Tables

**Figure:** Supplementary Content

